# P2X7 Variants in Pathophysiology

**DOI:** 10.3390/ijms25126673

**Published:** 2024-06-18

**Authors:** Anna Pegoraro, Marianna Grignolo, Luigia Ruo, Ludovica Ricci, Elena Adinolfi

**Affiliations:** Department of Medical Sciences, Section of Experimental Medicine, University of Ferrara, 44121 Ferrara, Italy; marianna.grignolo@unife.it (M.G.); luigia.ruo@unife.it (L.R.); ludovica.ricci@edu.unife.it (L.R.)

**Keywords:** P2X7, splice variants, SNP, cancer, inflammation, therapy resistance

## Abstract

P2X7 receptor activation by extracellular adenosine triphosphate (eATP) modulates different intracellular pathways, including pro-inflammatory and tumor-promoting cascades. ATP is released by cells and necrotic tissues during stressful conditions and accumulates mainly in the inflammatory and tumoral microenvironments. As a consequence, both the P2X7 blockade and agonism have been proposed as therapeutic strategies in phlogosis and cancer. Nevertheless, most studies have been carried out on the WT fully functional receptor variant. In recent years, the discovery of P2X7 variants derived by alternative splicing mechanisms or single-nucleotide substitutions gave rise to the investigation of these new P2X7 variants’ roles in different processes and diseases. Here, we provide an overview of the literature covering the function of human P2X7 splice variants and polymorphisms in diverse pathophysiological contexts, paying particular attention to their role in oncological and neuroinflammatory conditions.

## 1. Introduction

P2X7 is a member of the P2X plasma membrane purinergic receptor family (P2X1-7). It is a trimeric receptor composed of identical subunits containing intracellular short N-and long C-terminal tails, a large extracellular loop where ATP binds, and two transmembrane domains [1]. Receptor activation occurs when three ATP molecules interact with the binding pocket created at the interface between two subunits [2]. P2X7 differs from the other P2X subtypes for its double function. Transient stimulation induces cation channel activity, allowing the efflux of K^+^ and the influx of Na^+^ and Ca^2+^. A high ATP concentration or prolonged stimulation leads to the formation of a non-selective pore, enabling the passage of large molecules of up to 900 Da, such as YO-PRO-1 or ethidium bromide, inducing cell death [3]. Some evidence suggests that the pore derives from the dilatation of the channel itself or the interaction with other proteins. Also, the lipid composition can influence its formation; sphingomyelin facilitates it, while cholesterol interferes [4]. Although the mechanism is not fully demonstrated, the long C-terminal tail plays an essential role in macropore generation [4]. Different cell types express P2X7, including immune cells, exocrine/endocrine secretory cells, sympathetic/sensory and enteric neurons, astrocytes, oligodendrocytes, osteoblasts, osteoclasts, keratinocytes, fibroblasts, smooth muscles, and epithelial cells [5]. Generally, ATP is present at high concentrations intracellularly and at negligible levels in healthy tissues. However, ATP can be released in response to hypoxia, chemotherapy, radiotherapy, mechanical stress, and during necrotic and pyroptotic cell death [6]. In the extracellular milieu, it acts as a danger signal and activates P2X7, inducing various cellular responses. The best-characterized intracellular signaling is the activation of the NLRP3 inflammasome, leading to the maturation and release of the pro-inflammatory cytokines IL-1β and IL-18. Moreover, P2X7 stimulation can induce bone remodeling [7], proliferation [8], cell migration [9,10,11], vesicle secretion [12,13], and angiogenesis [8,14,15,16]. Extracellular ATP (eATP) accumulation occurs mainly in inflammatory and tumor microenvironments [17]. Indeed, P2X7 is associated with the development and progression of diverse diseases, including brain disorders [18,19], autoimmune diseases [20,21], blood malignancies [22], and cancer. [23,24,25].

## 2. P2X7 Splice Variants

The P2X7 gene consists of 13 exons [26], and, in 2005, Cheewatrakoolpong and colleagues reported the first eight variants identified in humans (P2X7A-P2X7H) obtained from alternative splicing events. P2X7A is the first variant, corresponding to the full-length protein with 595 amino acids. It derives from the translation of all 13 exons of the P2X7 gene. P2X7B, E, and G are truncated proteins in which an intron is inserted between exons 10 and 11, which introduce a premature stop codon. P2X7B was demonstrated to be functional, but the lack of the final 171 amino acids of the C-terminal tail and the insertion of 18 alternative amino acids following the second transmembrane domain did not allow for the formation of the macropore, thus maintaining only the channel activity. P2X7C and D lack exons four and five, respectively, and P2X7F does not have both exons four and eight. Variants G and H present a new exon named N3 inserted between exons two and three [27]. Other variants were found in subsequent studies. P2X7I derives from a point mutation in the splice site sequence of intron one, leading to a null allele [28]. P2X7J lacks exon eight, and it is the shortest variant without the C-terminal tail, with only one of the transmembrane domains and a part of the extracellular loop. It is non-functional and, when co-expressed with P2X7A, antagonizes its function [29]. P2X7L is missing part of the extracellular domain due to the deletion of exons seven and eight. When transfected in HEK 293 cells, P2X7L facilitated phagocytosis in response to ATP, but did not cause membrane blebbing, cell death, and channel and macropore opening. The presence of P2XL together with P2X7A halved the pore activity [30]. Finally, the specific epitope E200A characterizes the non-functional P2X7 (nfP2X7), which cannot form the cytotoxic pore [31]. 

## 3. P2X7 Splice Variants in Physiological and Pathological Conditions

The full-length protein P2X7A is considered the WT version of the human P2X7 receptor, since it is entirely functional and similar in structure to the receptors expressed in other species; for these reasons, it was the most studied over time. The discovery of the functional truncated forms and isoform combinations increased the curiosity about their role in processes generally associated with the full-length receptor. This section describes the current knowledge about P2X7 splice variants in physiological and pathological conditions. 

### 3.1. P2X7B in Cancer Growth, Metastasis, and Therapy Resistance

**Figure 1:**
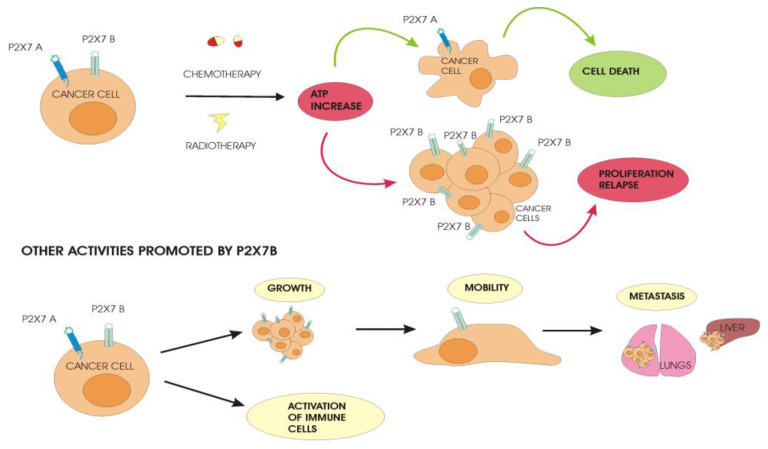
Differential behavior of cancer cells expressing P2X7A and P2X7B. Anticancer treatment with chemotherapy and radiotherapy induces the release of a high amount of ATP in the tumor microenvironment. Cancer cells expressing P2X7A in the presence of elevated levels of ATP undergo cell death due to the opening of the pore while P2X7B-expressing cells are not affected by the cytotoxic effect of ATP. The surviving cells proliferate and allow the relapse of the disease after treatment, promoting a more aggressive cancer that spreads and forms metastases in distant sites from the primary tumor.

The best-characterized human splice variant is P2X7B because, as mentioned before, it is still functional, although it only shows cation channel activity. When activated with eATP, it increases Ca^2+^ release from the endoplasmic reticulum (ER) and NFATc1 nuclear translocation, promoting cell proliferation in normal and low-serum conditions [32,33]. The growth-promoting activity is comparable to that of P2X7A, and both variants are often co-expressed in tissues that potentiate cell proliferation [32]. However, the separate transfection of P2X7A and B in Te85 osteosarcoma cells demonstrated that they could affect other cellular pathways differently. Te85 P2X7B increased osteoprotegerin levels, reducing mineralization compared to non-transfected cells and Te85 P2X7A [32]. Moreover, P2X7B promoted the migration of Te85 cells in vitro in a wound closure assay and was transfected in the osteosarcoma cells, while MNNG-HOS increased the capacity to form lung metastases when cells were injected into the mice’s tail veins, though it did not affect the growth of the primary tumor in the bone as compared to the non-transfected cells [33]. This metastasis-promoting effect was demonstrated in other cancer models. In prostate cancer, P2X7B expression was found, especially in patients with disease progression and bone metastasis. Similar results were observed in an in vivo model in which PC3 prostate cells, expressing the truncated P2X7, doubled the frequency of bone metastasis as compared to P2X7A-expressing cells, probably due to the increased release of the pro-inflammatory cytokine IL-6, which associates with cancer cell survival and angiogenesis [34]. The analysis of cDNA from patients with melanoma at different stages revealed a significantly increased expression of both P2XA and P2X7B in metastatic melanomas that spread to the brain, liver, and lungs, which are by far the most aggressive forms of this solid cancer [35]. Metastasis formation is often due to the cells’ ability to resist drugs [36]. Cancer cells develop different mechanisms to evade therapy, promoting disease relapse and dissemination. In acute myeloid leukemia (AML), we demonstrated that, in patients who experienced a return of the pathology after treatment, the expression of P2X7B significantly increased compared to newly diagnosed patients, while P2X7A expression significantly decreased. Instead, in remitting patients, both P2X7 variants were remarkably reduced [37]. HEK293 cells separately transfected with P2X7A and P2X7B, and helped us to clarify in vitro the role played by each variant when the cells were treated with daunorubicin [37], a drug that is usually administered in AML therapy and which is known to cause the release of a high amount of ATP [38]. HEK-P2X7A cells died more than the control cells, while the HEK-P2X7B cells were more resistant to chemotherapy’s toxicity. The elevated eATP, released following the daunorubicin treatment, induced the opening of the pore in HEK-P2X7A, facilitating the entry of the drug and intensifying its death-promoting function without having any effect on HEK-P2X7B [37]. The same behavior could be found in the AML patients’ cells. Chemotherapy can eliminate blasts expressing P2X7A through pore formation, while blasts with a high expression of P2X7B can survive and proliferate [37] (Figure 1).

Patients can differ from each other with respect to P2X7A and B expression in their cells, and this could be a reason why some heal completely and others have a relapse of the pathology after therapy. The cell death-promoting role of P2X7A and the protection of P2X7B upon chemotherapy was also demonstrated in neuroblastoma (NB), a neuronal cancer that affects the sympathetic peripheral nervous system [39]. In a recent study by Arnaud Sampaio and colleagues, the resistance of P2X7B-expressing cells to vincristine was correlated with several changes in NB cell features [39]. The expression of P2X7B in NB cells impaired autophagy and favored the epithelial–mesenchymal transition and drug efflux through the induction of the multidrug-resistance-associated protein 1 (MRP-1) pump expression. Moreover, cells expressing P2XB are resistant to retinoic acid, which acts as a negative modulator of the transporter MRP-1 [39]. Cells expressing P2X7A show the opposite effects: they are sensitive to retinoic acid, inducing a decrease in MRP-1 pump expression and preventing drug efflux [40]. Moreover, they mediate autophagy and exhibit an epithelial phenotype. These findings suggest that the prevalence of one of the two isoforms in neuroblastoma cells could be useful for treatment decisions using P2X7 agonists/antagonists in combination with other compounds like retinoic acid. The same research group has previously demonstrated that the pro-inflammatory factor bradykinin promotes NB progression and metastasis, upregulating P2X7B [41]. Bradykinin is released by damaged cells as ATP and modulates different pathways involved in carcinogenesis. These effects of bradykinin were blocked by the administration of P2X7 antagonists known to have the same pharmacological activity on both A and B isoforms [41]. These two studies on neuroblastoma reveal that other signaling systems could favor P2X7B-expressing cells. 

The release of large amounts of ATP from dying cancer cells also happens after radiotherapy, another treatment option for many cancers. In a study on glioblastoma (GBM), two cell lines that differed in the expression levels of P2X7A, but not of variant B, following irradiation showed opposite behaviors [42]. High-P2X7A GBM cells were sensitive to ATP-induced death, while low-P2X7A GBM cells were resistant to the cytotoxic effect of ATP. Further, days after irradiation, it was observed that a cell population characterized by the increased expression of P2X7B and decreased levels of P2X7A survived and grew [42]. The mechanism involved is supposed to be the same as seen in AML cells, where high ATP concentrations induced by radiotherapy lead to the opening of the cytotoxic pore in P2X7A-GBM cells, while the P2X7B-GBM cells resist and grow (Figure 1).

Interestingly, an increase in the release of cholesterol was observed following irradiation. This lipid negatively affects P2X7A macropore formation [4], and cells could use this further strategy to evade cell death. In another study, a small population of GBM stem cells resisting pharmacological treatment showed both P2X7A and B level upregulation and an increase in epithelial–mesenchymal transition factors, potentiating their aggressiveness [43]. The expression of P2X7B was also reported in lung adenocarcinoma (LUAD) [44]. Benzaquen and colleagues investigated the expression of P2X7 splice variants A, B, H, and J in cancer and immune-infiltrating cells from human LUAD patients. The expression of P2X7 A, B, H, and J was upregulated in immune cells and downregulated in tumors. Moreover, the expression of P2X7B was higher than that of the other variants, and was associated with a decrease in tumor-infiltrating T and B lymphocytes and an increase in myeloid cells. This study revealed the important role of P2X7B in modulating the immune infiltrate’s composition and demonstrated that the macropore function of P2X7A is decreased in its presence in the context of LUAD [45]. This last result contrasts with previous data on the positive modulation of the co-presence of P2X7A and B [32]. However, the authors evidenced the formation of a chimeric P2X7A-B protein, predominantly intracellularly retained, that could cause decreased receptor activity as a macropore [45].

### 3.2. Other Splice Variants in Cancer and Other Diseases

Although most of the published studies focused on P2X7B, interesting evidence also emerged on the other variants. P2X7J is present in cervical cells, mainly in cancer rather than in normal tissue, but in the presence of the full-length P2X7A, heterotrimerizes with it, preventing pore formation and cell death [46]. These data suggest that P2X7J may protect tumor cells from ATP-dependent cell death, making it a potential biomarker for cervical cancer [29] and a helpful indicator in choosing the best treatment options. nfP2X7 is exclusively expressed in cancer cells [47,48] and maintains only a channel function promoting cell survival in an ATP-rich condition [48]. The exposure of epitope E200 distinguishes it from the functional form expressed by healthy cells. This peculiarity led to the use of epitope E200-targeting antibodies as a new anticancer therapy. A phase I clinical trial on basal cell carcinoma (BCC) showed that nfP2X7-targeting antibodies were safe to use in BCC patients and caused a reduction in the lesion surface area [31]. Moreover, combining the anti-nfP2X7 molecule with chimeric antigen receptor (CAR) T cell immunotherapy strengthened its anticancer role [49]. Indeed, an nfP2X7-targeting CAR was generated and showed cytotoxic activity against multiple cancer lines in vitro and significantly reduced tumor growth in in vivo triple-negative breast cancer and prostate cancer models [49]. The role of P2X7 splice variants in diseases other than cancers is less well-known. P2X7A, B, H, and J isoforms were examined in the brains of patients with Huntington’s disease (HD), a neurodegenerative disorder characterized by a mutation in the huntingtin (HHT) gene [50]. P2X7A and B significantly increased in the striatum of HD patients compared to healthy patients; P2X7J did not change in the two populations, and P2X7H was not detected. Immunohistochemistry analysis showed that P2X7A expression was weak and restricted to the neurons’ cytoplasm and neuropil in the control striatum; in HD samples, it was also more intense and diffuse in the neuron processes. P2X7B is localized in the neuronal processes and cytoplasm in both control and HD brains, but the expression is more intense in the second group [50].

## 4. P2X7 Single-Nucleotide Polymorphisms (SNPs) 

Numerous single-nucleotide polymorphisms (SNPs) were identified in the P2X7 gene; some of them were non-synonymous and introduced a mutation in amino acid sequences (Table 1). Three SNPs are common in humans, with a minor allele frequency >25% [51]. The 489 C>T (rs208294) and the 835 G>A (rs7958311) are characterized by the substitution of the histidine in positions 155 and 270 of the extracellular domain with tyrosine and arginine, respectively. The 1068 G>A (rs1718119) has a threonine instead of alanine at position 348 in the second transmembrane domain. Four SNPs showed a minor allele frequency of >5% [51]. Three were localized in the carboxy-terminal tail; the glutamic acid in positions 496 and 460 was substituted by an alanine (1513 A>C, rs3751143) and arginine (1405 A>G, rs2230912), respectively, while the threonine in position 357 was changed with serine (1096 C>G, rs2230911). The 370 T>C (rs17525809) yielded an alanine-to-valine change in position 76 in the extracellular domain. The 1068 G>A and 489 C>T SNPs were shown to be gain-of-function variants [52], while the other ones mentioned above were associated with loss-of-function variants. The other SNPs reported in the literature showed a minor frequency between 5% and 0.5% [51]. They include the loss-of-function SNPs 474 G>A (rs28360447), 946 G>A (rs28360457), 1729 T>A (rs1653624), and 853 G>A (rs7958316). The 1729 T>A is located at the terminal C-tail in position 568, where the isoleucine was substituted with asparagine. The others localized on the extracellular domain, as follows: 474 G>A induced the change in glycine at position 150 with arginine, while the 853 G>A and 946 G>A were characterized by the replacement of arginine at positions 276 and 307 with histidine and glutamine, respectively [53]. 

## 5. P2X7 SNPs in Physiological and Pathological Conditions

In human diseases, the expression of P2X7 SNPs has largely been investigated, and many studies have demonstrated that the loss- or gain-of-function variants are related to different pathologies (Table 1). Some SNPs influence the activity of the receptor as a channel or pore, leading to modifications of the cell behavior. For example, monocytes and B and T lymphocytes carrying the 1729 T>A mutation showed a very low or absent P2X7 function. This mutation reduced not only the activity of the receptor but also its trafficking on the cell surface [54]. On the contrary, in human monocytes treated with LPS, the 1068 G>A mutation increased the release of the pro-inflammatory cytokine IL-1β. This finding was associated with patients with hyperuricemia with a higher risk of developing gout [55]. This mutation is also relevant for β-cell function and glucose regulation. Subjects with this variant are more insulin-sensitive and respond better during energy demand. Cells can secrete ATP as a danger signal and enhance the glucose metabolism in the presence of gain-of-function P2X7 SNPs on β-cells [56]. The co-presence of the 1068 G>A and 1405 A>G mutations form a gain-of-function receptor, increasing the mobilization of the healthy human hematopoietic stem cell CD34^+^ following the administration of granulocyte–colony stimulating factors (G-CSFs) [57]. 

### 5.1. P2X7 SNPs in Oncological Conditions

Most of the studies covering the role of P2X7 SNPs in oncology are correlation analyses [46]. The 1513A>C is probably the most studied SNP, leading to a non-functional receptor formation. In B-chronic lymphocyte leukemia (B-CLL), contrasting data were published about this SNP. It has been associated with cancer-promoting [58] and protective functions [59]. Instead, other studies have found no association between the 1513A>C mutation and B-CLL [60,61]. However, these investigations were carried out in some familiar cases and at different stages of the disease without taking into account any other variants of P2X7 [22]. There is no difference between healthy subjects and patients with papillary thyroid cancer for the incidence of this SNP. Nevertheless, patients affected by the follicular subtype of papillary thyroid cancer did express increased levels of the 1513A>C SNP [62]. The authors of this research suggested that a defective P2X7 SNP can cause a dysregulation of the host immune system, favoring tumor growth [62]. A similar mechanism was proposed for breast cancer, in which the decreased activity of the receptor on the monocytes reduced the affinity for ATP and the secretion of IL-1β from immune cells [63]. In this study, breast cancer cells were treated with anthracycline, which, as with other chemotherapeutic agents such as oxaliplatin or dauno- and doxorubicin, induced the release of high amounts of ATP, which activated the NLRP3 inflammasome and the production of IL-1β, inducing immunogenic cell death (ICD) [17,38]. Therefore, the authors suggest that the P2X7 polymorphism 1513A>C can reduce the effect of anthracycline by dampening ICD [63]. Various other studies have taken into account the association of more than one P2X7 polymorphism to cancer- and tumor-related symptoms. For example, a study considering cancer-related pain demonstrated that, after mastectomy, patients with breast cancer expressing the gain-of-function 489 C>T SNP suffered more than those expressing the loss-of-function 835 G>A SNP [64]. This finding suggests that blocking the P2X7 receptor could provide a new strategy to alleviate cancer pain. A case-control study conducted on patients with hepatocellular cancer from the Chinese Han population proposed that the P2X7 polymorphisms 1513A>C and 946 G>A contribute to changing cellular microenvironment conditions. Specifically, the 1513A>C SNP affects protein expression, while the 946 G>A SNP negatively influences cell apoptosis and the ATP interaction. On the contrary, the 1068 G>A SNP seems to be protective, as it promotes immune responses against cancer [65].

Another set of experiments showed that, in pancreatic ductal adenocarcinoma, the 474 G>A and 853 G>A SNPs decreased and increased the risk of cancer development, respectively. Moreover, cancer cells expressing the 474 G>A variant showed a reduction in the migration capacity, suggesting that this variant might have a protective role against metastasis [66]. 

### 5.2. P2X7 SNPs in Mood Disorders 

Mood disorders, including depression, suicidal behavior, bipolar disorder, and anxiety, have been, by far, the conditions in which P2X7 SNPs have been explored the most in-depth. Several papers, reviews, and meta-analyses have been conducted covering this subject, and we refer the readers to these overviews for a more comprehensive understanding of the field [67,68,69,70,71]. Data on SNPs, together with preclinical tests carried out on animal models, have led to the development of new brain-permeant P2X7 antagonists such as JNJ-54175446, which was also tested in patients with major depressive disorder [72]. This trial, while demonstrating minor side and adverse effects of the P2X7 antagonist, failed to prove any amelioration of mood-related symptoms, with the exception of anhedonia in major depressive disorder patients exposed to total sleep deprivation for 36 h [72]. However, the availability of brain-permeant P2X7 blockers opens the way to the treatment of many other diseases of the central nervous system, including other mood disorders, but also of neurodegenerative and neuroinflammatory conditions [73].

### 5.3. P2X7 SNPs in Other Diseases

The P2X7 receptor is central for many other diseases with an immune/inflammatory correlation, where its polymorphic variants have been suggested to play a role. 

In Alzheimer’s disease, the 1513A>C nucleotide change seems to be protective, as a decrease in the frequency of allele C and an increase in both AA and AC genotypes in patients was observed as compared to healthy individuals, suggesting that a fully functional receptor is necessary in the Alzheimer’s etiology [74]. In accordance with these data, the gain-of-function variant 489 C>T SNP is also associated with Alzheimer’s disease [75]. 

A combination of the 1068 G>A and 1405 A>G mutations was correlated with the progressive disability of patients with multiple sclerosis [76]. The 1045 A>G SNP was also identified as a potential biomarker to identify patients at risk of developing ocular toxoplasmosis [77]. The gain-of-function 489 C>T SNP was also associated with systemic Lupus erythematosus [78], HHV-6A infection, and related infertility [79]. The loss-of-function variant 1513 A>C was upregulated in osteoporosis [80], tuberculosis [81,82], and cardiovascular diseases [83]. In osteoporosis, allele C impaired ATP-induced apoptosis in osteoclasts, which are central to maintenance, repair, and bone remodeling [84]. 

## 6. Conclusions

Since the P2X7 receptor modulates many intracellular pathways, alterations in its structure, expression, and function can cause substantial changes in cell behavior, leading to the onset of inflammatory-related diseases and cancer. Many studies revolving around the P2X7 splice or partially functional variants have focused on liquid and solid cancers, suggesting that therapies targeting these receptors’ isoforms could be helpful in preventing cancer growth, dissemination, and resistance to traditional anti-tumoral therapies [36]. However, more effort is required to develop molecules able to block exclusively specific receptor variants. Moreover, the ample variation in the P2X7 genotype can be exploited in tailored diagnostic and therapeutic approaches. However, most of the studies on the P2X7 SNPs present some limitations. They often only provide an association analysis with the diseases obtained from small sample sizes or specific closed populations. In the future, we will need appropriate pathology models to validate the results using experimental protocols.

## Figures and Tables

**Table 1:** Main single-nucleotide polymorphism (SNP) substitutions of the human P2X7 receptor and related pathological conditions. MAF: minor allele frequency; BC: base change; AAC: amino acid change; HHV-6A: human herpesvirus 6A; AD: Alzheimer’s disease, B-CLL: B-chronic lymphocytic leukemia; BD: bipolar disorder.

db SNP ID	MAF	BC	AAC	P2X7 Function	Pathological Conditions
rs208294	>25%	489 C>T	H155Y	gain	breast cancer, HHV-6A infection, infertility, AD, systemic lupus erythematosus
rs7958311	>25%	835 G>A	H270R	loss	breast cancer
rs1718119	>25%	1068 G>A	A348T	gain	Hepatocellular cancer, depression, mania, multiple sclerosis, acute bipolar disorder, gout
rs3751143	>5%	1513 A>C	E496A	loss	B-CLL, breast cancer, papillary thyroid and hepatocellular cancers, AD, BD, osteoporosis, tuberculosis, cardiovascular disease
rs2230912	>5%	1405 A>G	Q460R	loss	Sleep alteration, depression, mania, multiple sclerosis, acute BD, ocular toxoplasmosis
rs2230911	>5%	1096 C>G	T357S	loss	na
rs17525809	>5%	370 T>C	V76A	loss	na
rs28360447	5–0.5%	474 G>A	G150R	loss	Pancreatic ductal adenocarcinoma
rs28360457	5–0.5%	946 G>A	R307Q	loss	Hepatocellular cancer
rs1653624	5–0.5%	1729 T>A	I568N	loss	na
rs7958316	5–0.5%	853 G>A	R276H	loss	Pancreatic ductal adenocarcinoma
